# Mechanism of Sugarbeet Seed Germination Enhanced by Hydrogen Peroxide

**DOI:** 10.3389/fpls.2022.888519

**Published:** 2022-04-25

**Authors:** Chenggen Chu, Rachael Claire Poore, Melvin D. Bolton, Karen K. Fugate

**Affiliations:** Sugarbeet and Potato Research Unit, Edward T. Schafer Agricultural Research Center, Agricultural Research Service, United States Department of Agriculture, Fargo, ND, United States

**Keywords:** sugar beet, seed dormancy, RNA-Seq, gene expression, differentially expressed gene, transcript analysis

## Abstract

Seed germination is a critical first stage of plant development but can be arrested by factors including dormancy and environmental conditions. Strategies to enhance germination are of interest to plant breeders to ensure the ability to utilize the genetic potential residing inside a dormant seed. In this study, seed germination in two sugarbeet (*Beta vulgaris ssp. vulgaris* L.) lines F1004 and F1015 through incubating seeds in hydrogen peroxide (H_2_O_2_) solution was improved over 70% relative to germinating seeds through water incubation. It was further found that low germination from water incubation was caused by physical dormancy in F1015 seeds with initial seed imbibition blocked by the seed pericarp, and physiological dormancy in F1004 seeds with germination compromised due to the physiological condition of the embryo. To identify genes that are differentially expressed in response to cellular activities promoted by H_2_O_2_ during overcoming different type of dormancies, an RNA-Seq study was carried out and found H_2_O_2_ treatment during germination accelerated the degradation of seed stored mRNAs that were synthesized before or during seed storage to provide protections and maintain the dormant state. Comparison of transcripts in H_2_O_2_-treated seeds between the two sugarbeet lines identified differentially expressed genes (DEGs) that were higher in F1004 for alleviating physiological dormancy were known to relative to gene expression regulation. The research established that H_2_O_2_ overcomes both physical and physiological dormancies by hastening the transition of seeds from dormancy into germination. More DEGs related to gene expression regulation were involved in relieving physiological dormancy which provides new knowledge about the role of exogenous H_2_O_2_ as a signaling molecule for regulating gene activities during germination. Moreover, the protocol using H_2_O_2_ to promote germination will be useful for rescuing plant germplasms with poor germination.

## Introduction

Seed germination is the initial developmental stage responsible for producing plants of the next generation to maintain the germplasm and multiply individuals of the species. From initial water uptake to the development of the embryonic axis, the germination process involves numerous events including protein hydration, subcellular structural changes, respiration rate increases, macromolecular syntheses, cell elongation and division, and many other metabolic activities (Bewley and Black, 1994). Seed germination, therefore, is a very complex process and can be influenced by factors of extrinsic (water, temperature, light, and oxygen) and intrinsic (mainly the dormancy due to physiological condition within seed) natures (Raven et al., 2005). Adjusting extrinsic factors to provide more favorable conditions may not always lead to successful germination if seeds are in a dormant state (Baskin and Baskin, 2004). Consequently, breaking seed dormancy by stimulating key processes related to germination often becomes vital for seedling establishment and proliferation in the new generation.

Seed dormancy is a mechanism that evolved to slow down or inhibit germination under unsuitable conditions to help plant species survive under adverse conditions. In general, seed dormancy includes exogenous physical dormancy due to physical restrictions caused by the seed coat as well as endogenous physiological dormancy caused by physiological conditions within the embryo itself (Baskin and Baskin, 2004). Physical dormancy can be broken naturally during seed imbibition or artificially through scarification, dry heat, fire, or application of hot water, acid, or other chemicals to open seed coats and allow seeds to take up the water and oxygen needed for germination (Emery, 1988). In contrast, overcoming physiological dormancy, which arises from the presence of germination inhibitors or other unknown physiological factors, may require a biochemical trigger to initiate the germination process (Keeley, 1991).

Hydrogen peroxide (H_2_O_2_) has been shown to stimulate germination (Fontaine et al., 1994; Katzman et al., 2001; Zeinalabedini et al., 2009), but the mechanism by which it acts is largely unknown. H_2_O_2_ and other reactive oxygen species (ROS) have been detected in seeds during water imbibition and the early stages of germination (Schopfer et al., 2001; Bailly, 2004; El-Maarouf-Bouteau and Bailly, 2008; Kranner et al., 2010; Zhang et al., 2014; Wojtyla et al., 2016). H_2_O_2_ accumulation has also been shown to be a signaling cue for regulating seed dormancy and germination (Bailly et al., 2008; Bailly, 2019). According to the “oxidative window” hypothesis proposed by Bailly et al. (2008), only a critical range of ROS concentrations alleviates dormancy while levels below or above the critical range impair germination. Verma et al. (2015) postulated that stored proteins were oxidatively modified by H_2_O_2_ and ROS, and the modified proteins were then recognized by storage organs as signals to mobilize nutrients for the rapid growth that is needed for germination. Oxidation induced alterations in enzymatic and binding properties of proteins have been observed by Davies (2005), and protein oxidations were also observed during germination of pea (Barba-Espín et al., 2010) and Arabidopsis (Job et al., 2005). Therefore, selective oxidation of the seed proteome and transcriptome remodeling by H_2_O_2_ and ROS may play a role in releasing dormancy to start germination (Diaz-Vivancos et al., 2013).

Another possible mechanism by which H_2_O_2_ promotes germination is *via* crosstalk with phytohormones such as abscisic acid (ABA) and gibberellic acid (GA) which have known roles in maintaining and releasing dormancy, respectively (Finch-Savage and Leubner-Metzger, 2006; Finkelstein et al., 2008; Nambara et al., 2010; Weitbrecht et al., 2011; Graeber et al., 2012; Rajjou et al., 2012; Diaz-Vivancos et al., 2013; Golldack et al., 2013; Rodríguez et al., 2015; Shu et al., 2016; Wojtyla et al., 2016; Ishibashi et al., 2017; Carrera-Castaño et al., 2020). For example, ROS and H_2_O_2_ concentrations increased with addition of GA in germinating seeds of radish (Schopfer et al., 2001) and Arabidopsis (Liu et al., 2010; Lariguet et al., 2013), thereby changing oxidative conditions and enhancing germination. Bahin et al. (2011) proposed that H_2_O_2_ affects GA signaling by modulating phytohormone balance and initiating germination in barley, which was later supported by Lariguet et al. (2013) as they observed that oxidative levels were enhanced by GA treatment but declined with ABA treatment in Arabidopsis seeds. Other research has indicated that H_2_O_2_ may release the inhibitory effect of ABA to initiate germination in Arabidopsis (Müller et al., 2006, 2007) and lettuce (Zhang et al., 2014).

H_2_O_2_ and ROS have also been shown to be signal molecules that regulate expression of germination-related genes. Lariguet et al. (2013) suggested that H_2_O_2_ may regulate the expression of genes encoding enzymes that hydrolyze the testa and endosperm of Arabidopsis seeds. Bray and West (2005) and Weitbrecht et al. (2011) proposed that ROS induce the expression of DNA ligase for DNA repair during Arabidopsis seed imbibition since the excess ROS can induce DNA damage and affect the accumulation of transcripts. Also, H_2_O_2_ and ROS have been shown to alter seed physiology during germination, thus affecting gene transcription (Weitbrecht et al., 2011) and the degradation of stored mRNAs in seeds (El-Maarouf-Bouteau et al., 2015). Overall, information currently available regarding the effect on gene expression and other cell activities when breaking seed dormancy through H_2_O_2_ and ROS is extremely limited.

Sugarbeet (*Beta vulgaris* ssp. *vulgaris* L.) is a relatively new crop with economic importance worldwide. Sugarbeet seeds are woody fruits commonly called seedballs. Inside each seedball, one (monogerm) or up to five (multigerm) kidney-shaped seeds are tightly wrapped in a woody pericarp that is composed of impervious sclerenchyma cells (Ignatz et al., 2019). The inner dense layer of pericarp restricts water and oxygen uptake by the enclosed seeds (Coumans et al., 1976; Richard et al., 1989; Tohidloo et al., 2015; Chomontowski and Podlaski, 2020), but the basal pore at the bottom of the pericarp is comprised of loose cells that provide a site of entry for water and oxygen. Hermann et al. (2007) analyzed the germination of sugarbeet fruit and isolated individual seed using selected plant hormone treatments and found that ethylene or the ethylene precursor, 1-aminocyclopropane-1-carboxylic acid (ACC), promoted radicle emergence of both fruits and the isolated seeds, while ABA inhibited radicle emergence from isolated seeds but not fruits. They thus proposed that the pericarp may block entry of exogenous ABA and restrict leaching of endogenous ACC. In other research, McGrath et al. (2000) used H_2_O_2_ solutions to discriminate vigor of seeds from various sugarbeet lines or seed lots since H_2_O_2_ stimulated germination and thus provided the optimal germinating conditions. These studies confirmed that ABA inhibits and ethylene and H_2_O_2_ promote sugarbeet germination. However, gene expression regulation during the release of seed dormancy toward germination remains unknown.

While germinating seeds of sugarbeet germplasms that were cold stored for many years, we encountered many lines with poor germination when seeds were incubated in water. Our preliminary tests indicated that incubating these seeds in a H_2_O_2_ solution improved germination. Research, therefore, was conducted to develop an efficient protocol of using H_2_O_2_ to enhance sugarbeet germination. Additionally, gene expression was compared between sugarbeet lines and between H_2_O_2_- and water-treated seeds to understand the mechanisms by which H_2_O_2_ promotes germination in seeds whose germination is restricted by physical or physiological dormancy.

## Materials and Methods

### Plant Materials

Seedballs from two sugarbeet lines F1004 (PI 590763) and F1015 (PI 605413) were used in this research. F1004 was released in 1984 and developed from mass selection for resistance to storage rot (Campbell and Bugbee, 1984). F1015 was released in 1996 as the first publicly available sugarbeet germplasm line with sugarbeet root maggot (*Tetanops myopaeformis* von Röder) resistance (Campbell et al., 2000). Both lines produce multigerm seedballs. Seedballs of F1004 and F1015 were stored in the USDA-ARS sugarbeet genetics laboratory in Fargo, ND, United States at 4°C and 40% relative humidity for 24 and 7 years, respectively. Seedballs of F1004 were polished by manually rubbing seedballs to remove the out loose layer of the pericarp, while seedballs of F1015 were raw fruits without any treatment. Preliminary tests indicated that germination of both lines was very low when seeds were germinated by incubating in distilled water or wetted soil. In addition, a set of 50 additional sugarbeet lines that was cold stored in the same lab for 10–37 years was used for validating the protocol of using H_2_O_2_ to enhance germination.

### Germination Experiments

Germination experiments were designed to determine the optimum length of time to incubate seeds in a H_2_O_2_ solution and evaluate the effects of environmental conditions such as seed hydration, temperature, and light on H_2_O_2_-stimulated germination. Also, germination of seeds that were manually isolated from seedballs was performed to identify the nature of seed dormancy for the two sugarbeet lines.

For determining the optimum length of time for incubating seeds in a H_2_O_2_ solution, five H_2_O_2_ treatments along with a water control were used. For each treatment, seedballs were incubated in a 1% H_2_O_2_ solution for 1, 2, 3, 4, or 7 days, then incubated in distilled water for the remainder of the 8-days period. Germination was evaluated daily. After 8 days, ungerminated seeds from each treatment were incubated for an additional 2 days in distilled water, and seeds were considered as having no germinability if no germination occurred at this time. Each treatment included three replications for each line with each replication comprised of 100 seedballs in a 9-cm diameter petri plate. Therefore, a total of 300 seedballs from each line was tested in each treatment. All petri plates were incubated at room temperature under fluorescent lights with light intensity around 11.9 μmol m^–2^ s^–1^ for approximately 9 h per day. On the first day, seedballs in each plate were incubated in 15 mL 1% H_2_O_2_ or distilled water, depending on the treatment. On each of the following days, incubating solution was decanted off and seedballs were washed three times with distilled water. To maintain treatment conditions but leave seedballs partially uncovered by liquid, 5 mL fresh 1% H_2_O_2_ or distilled water was added to each plate according to the treatment. Seedballs were considered germinated when radicles emerged and percentages of germinated seedballs were recorded and compared among treatments.

To determine the effect of seed hydration on H_2_O_2_ enhancement of germination, five treatments were used with seedballs incubated in distilled water for 0, 1, 2, 3, or 4 days prior to treatment with a 1% H_2_O_2_ solution. The effect of light on germination was tested using seedballs incubated either in water or H_2_O_2_ solution for 8 days at room temperature in the dark or under the natural light obtained through a window, with an approximate light intensity of 26.8 μmol m^–2^ s^–1^ for about 12 h per day. To test the influence of temperature on germination in H_2_O_2_ solution, two treatments were used. For the first treatment, seedballs were incubated in a 1% H_2_O_2_ solution at 4°C for 3 days and then incubated in H_2_O_2_ for an additional 4 days at room temperature; for the second treatment seedballs were incubated in 1% H_2_O_2_ at room temperature throughout the experiment. All experiments utilized three replicate petri plates per treatment for each of the two lines with each replicate comprised of 100 seedballs. Seedballs were rinsed with water each day and fresh 1% H_2_O_2_ solution or distilled water were added as described above. For validating the H_2_O_2_ germination protocol, two plates each containing 100 seedballs, were used for H_2_O_2_ treatment and water control, respectively, for each of 50 sugarbeet lines described above. Seeds were rinsed daily as described for germination experiments, and germination percentage was recorded on the 8th day. Analysis of variance (ANOVA) was conducted using the *aov* function in R^1^ to test the significance of each factor affecting germination, and the *LSD.test* function in R^2^ was used to calculate LSDs (least significant differences) at *P* < 0.05 for each treatment in each line to compare the significance of treatments on germination. Analyses of correlation, *t*-tests, and standard deviation calculations were conducted using functions of Microsoft Excel.

To determine if endogenous physiological or exogenous physical dormancy was responsible for the low germination of the two lines when seedballs were germinated in water, individual seeds were carefully isolated from seedballs. A hammer was used to slightly crack the pericarp, and seeds were carefully excised from broken seedballs. The isolated seeds were visually inspected and any seeds with visible damage were excluded. A total of 50 isolated seeds from each line were placed in a 9-cm diameter petri plate and incubated in 5 mL of distilled water. Seeds were washed daily by decanting the old rinse solution and washing seeds with water. Germination was checked daily.

### RNA Extraction, Library Construction and Sequencing

RNA-sequencing (RNA-seq) was used to analyze gene transcripts during seed imbibition and up to germination. From germination tests using H_2_O_2_ solution it was determined that seeds very likely had lost viability if they showed no germination after 8 days in H_2_O_2_ solution. Samples for RNA extraction thus were prepared using an 8-days period with seedballs treated with 1% H_2_O_2_ solution or distilled water using two replications for each line. A total of 64 plates (two lines × two treatments × two replications × 8 days) were used with each plate comprised of 100 seedballs. Eight plates (two lines × two treatments × two replications) were sampled each day. Since the purpose of this experiment was to detect genes expressed during seed imbibition and metabolic reawakening under H_2_O_2_ or water treatment, germinated seedballs with roots that extended out of the seed coat were excluded to minimize mRNAs that were transcribed in the root tissue that were irrelevant to germination. Therefore, seedballs with emerged radicles were counted each day and removed from each plate before collecting samples for RNA extraction. Collected samples were immediately frozen in liquid nitrogen and stored at –80°C until processing.

Total RNA was extracted and purified using a Qiagen-RNeasy Micro Kit according to the manufacturer’s instructions. Based on a preliminary test that almost no RNA was extracted from samples using seed pericarp only (data not shown), the whole seedballs thus were used for total RNA extraction. An Agilent 2100 Bioanalyzer (Agilent RNA 6000 Nano Kit) was used to determine RNA concentration, RIN value, the 28S/18S ratio, and fragment length distribution, and a NanoDrop 2000 spectrophotometer (Thermo Scientific, Inc., Waltham, MA, United States) was used to assess the purity of the RNA samples. To prepare sequencing libraries, mRNAs were isolated using poly-T oligo-conjugated magnetic beads, and cDNA was synthesized using reverse transcriptase Super Script II (Invitrogen, Waltham, MA, United States) at 42°C and random primers after RNA fragmentation. Second strand cDNA was synthesized using DNA Polymerase I and RNase H. The cDNA fragments were modified by the addition of A-tails and ligation of sequencing adapters and purified and enriched through PCR amplification. After a quality check, samples were pooled, and single strand DNA circles (ssDNA circles) were made. DNA nanoballs (DNBs) were generated by rolling circle replication (RCR) of ssDNA circles and the DNBs were loaded into patterned nanoarrays. Sequence data comprised of 100-bp pair-end reads were collected on a DNBseq platform using the combinatorial probe-anchor synthesis (cPAS) method (Fehlmann et al., 2016).

### Transcriptome and Gene Expression Level Analysis

Raw reads were filtered by removing adapters and trimming low quality bases (Phred score < 20) at the end of reads using the program SOAPnuke^3^. Filtered reads were aligned to the sugarbeet genome sequence, RefBeet v1.2.2 (Dohm et al., 2014; available from NCBI^4^) using the tool HISAT2 (Hierarchical Indexing for Spliced Alignment of Transcripts) (Kim et al., 2015). Transcript assemblies were generated using the computer package StringTie (Martin and Wang, 2011; Pertea et al., 2015) followed by use of Cuffcompare (Trapnell et al., 2012) to compare reconstructed transcripts to the reference annotation. The computer package CPC (Kong et al., 2007) was used to predict novel transcripts and merge novel transcripts with the reference genome to get a complete transcript reference. Transcript abundance was normalized to FPKM (fragment per kilobase of transcript per million reads mapped) values which were calculated using the computer package RSEM (Li and Dewey, 2011). Differentially expressed genes (DEGs) were detected using PoissonDis that is based on the Poisson distribution (Audic and Claverie, 1997) and limited to DEGS with a fold change > 2 and a false detection rate (FDR) at *P* < 0.001. Comparisons of gene expression were performed between treatments (H_2_O_2_ treatment vs. water control) to identify transcriptional changes in seed due to H_2_O_2_ and between the two sugarbeet lines (F1004 vs. F1015) to reveal transcriptional differences corresponding to different types of seed dormancy.

### Gene Functional Assignment and Expression Level Comparison

Gene ontology (GO) analysis was done according to Young et al. (2010) and GO functional enrichment determined using *phyper*, a function of R^5^. Gene functional annotation used the gene IDs and annotations in RefBeet (Dohm et al., 2014). For novel genes that did not align with any annotated genes in the sugarbeet genome, DNA sequences were compared with those stored in the NCBI GenBank database^6^ to infer gene function based on sequence similarity with a critical expectation value (*E* value) threshold set at 1.0 × 10^–5^ (Altschul et al., 1997). Pathway analysis of DEGs was conducted through KEGG (Aoki-Kinoshita and Kanehisa, 2007). Online protein databases (such as UniProt, SwissProt, and TrEMBL) were also searched using amino acid sequences or protein names as queries to identify possible biological processes or pathways of the proteins encoded by DEGs.

## Results

### Effect of H_2_O_2_ Treatment Time on Germination

In the water control, none of F1015 but 7.3% of F1004 seedballs germinated (Table 1). The 1-day treatment in 1% H_2_O_2_ solution had almost no effect on enhancing germination when compared with the water control (Table 1). This suggests that during the first day of incubating seedballs in H_2_O_2_ solution or water, only the pericarp rather than the enclosed seeds, were taking up water, and thus seed imbibition was unlikely to start on the first day. As the days of H_2_O_2_-treatment increased, germination of both lines increased by about 10% in the 2-day H_2_O_2_ treatment and by another 30% in each of the 3- and 4-days H_2_O_2_ treatments (Table 1). The highest germination percentage occurred in the 7-days H_2_O_2_ treatment in both lines with germination reaching 80.0% in F1004 and 86.7% in F1015 (Table 1), an increase of about 10% in F1004 and 20% in F1015 compared to the percentage observed in seedballs of the 4-days H_2_O_2_ treatment. This suggests that few seedballs require five or more days incubating in H_2_O_2_ solution to promote germination. The ungerminated seeds from the 7-days H_2_O_2_ treatment were likely unviable since they were mostly unable to germinate even after extending the H_2_O_2_ treatment time. Therefore, incubating seeds in H_2_O_2_ solution enhanced sugarbeet germination with the highest germination percentage achieved using a 7-days treatment.

**TABLE 1:** Germination induced by incubating seedballs of sugarbeet lines F1004 and F1015 in 1% H_2_O_2_ solution for varying durations under fluorescent lights at room temperature.

Treatment^a^	Germination percentage^b^	
	F1004	F1015
Water control	7.3 ± 1.1 a	0.0 ± 0.0 a
H_2_O_2_ 1-day	7.6 ± 1.1 a	0.7 ± 0.6 a
H_2_O_2_ 2-days	17.6 ± 3.2 b	11.0 ± 2.0 b
H_2_O_2_ 3-days	43.7 ± 8.3 c	39.0 ± 2.0 c
H_2_O_2_ 4-days	71.3 ± 2.1 d	65.3 ± 2.5 d
H_2_O_2_ 7-days	80.0 ± 5.3 e	86.7 ± 10.8 e

*^a^Water control means seedballs were incubated in the distilled water for the 8-days duration of the experiment, treatments of H_2_O_2_ 1-, 2-, 3-, 4-, and 7-days indicate that seedballs were incubated in 1% H_2_O_2_ solution accordingly for 1, 2, 3, 4, and 7 days followed by incubating in distilled water for the remaining days of the experiment.*

*^b^Numbers followed by the same letter within each column are not significantly different from one another at the 0.05 level of probability.*

### Effects of Environmental Factors on Germination Induced by H_2_O_2_

For evaluating the effect of seed hydration on germination in H_2_O_2_ solution, seedballs were preincubated in water for 1–4 days to achieve different levels of hydration. Overall, all water-preincubated seedballs germinated more poorly in both lines, with the reduction in germination more severe as seedballs were incubated for longer durations in water (Table 2). Germination of seedballs preincubated in water for 1 day followed by H_2_O_2_ treatment was reduced by approximately 30% compared to seedballs that receive no water pretreatment. A further 20–30% reduction in germination was observed with 2 and 3 days of water preincubation. Seedballs preincubated in water for 4 days followed by H_2_O_2_ treatment showed a similar germination percentage to the water control (Table 2), indicating that H_2_O_2_ treatment did not enhance germination if seeds were preincubated in water for 4 or more days. Therefore, increasing hydration in seeds did not improve germination under H_2_O_2_ treatment.

**TABLE 2:** Seedball germination to determine effects of hydration, light, and temperature on germination induced by H_2_O_2_ solution.

Treatment	Germination percentage^a^	
	F1004	F1015
(1) Different hydration levels under lab light at room temperature		
Seedballs in H_2_O_2_ solution for 7 days	77.0 ± 10.0 a	81.3 ± 5.0 a
Seedballs 1 day in water, then 6 days in H_2_O_2_	46.7 ± 2.5 b	55.0 ± 2.6 b
Seedballs 2 days in water, then 5 days in H_2_O_2_	30.3 ± 2.1 c	34.3 ± 2.5 c
Seedballs 3 days in water, then 4 days in H_2_O_2_	13.3 ± 1.5 d	4.7 ± 2.3 d
Seedballs 4 days in water, then 3 days in H_2_O_2_	9.3 ± 1.2 d	0.0 ± 0.0 d
(2) With or without light at room temperature^b^		
Water control under natural light from window	9.3 ± 2.9 a	0.3 ± 0.6 a
Water control under dark	21.0 ± 7.9 b	0.7 ± 1.2 a
H_2_O_2_ treated under natural light from window	74.3 ± 0.6 c	86.7 ± 6.5 b
H_2_O_2_ treated under dark	80.7 ± 5.1 c	82.0 ± 8.2 b
(3) Different temperature under dark		
Water control with 3 days at 4°C, then room temperature	4.3 ± 0.6 a	0.0 ± 0.0 a
Water control at room temperature	19.3 ± 3.1 b	0.7 ± 1.2 a
H_2_O_2_ treated with 3 days at 4°C, then room temperature	12.0 ± 3.5 c	47.7 ± 7.4 b
H_2_O_2_ treated at room temperature	72.0 ± 5.6 d	87.3 ± 4.9 b

*^a^Numbers followed by the same letter within each column under each treatment are not significantly different from one another at the 0.05 level of probability.*

*^b^Light intensity of natural light close to window is approximately 26.8 μmol m^–2^ s^–1^.*

Dark conditions increased germination by about 10% in water-incubated F1004 seedballs but had no effect on water-incubated F1015 seedballs. When using H_2_O_2_ treatment to induce germination, dark conditions increased germination of F1004 seedballs by 6.4% but had no effect on germination of F1015 seedballs (Table 2). Therefore, dark conditions are limitedly effective in increasing germination when utilizing a H_2_O_2_ treatment, with its effectiveness dependent on genotype.

Three days of cold treatment greatly decreased germination of both lines when germinated in H_2_O_2_ solution or water (Table 2). Only 4.3% of cold-treated F1004 seedballs germinated in the water control, which was significantly reduced from the 19.3% germination observed in the water control at room temperature. With the H_2_O_2_ treatment, germination of seedballs that received the 3-days cold treatment was 12.0% in F1004 and 47.7% in F1015. These germination percentages were much lower than those (80.7 and 82.0% in F1004 and F1015, respectively) obtained with seedballs that were not cold treated (Table 2). Therefore, no cold treatment was needed when using H_2_O_2_ to induce germination. Overall, these results suggest that using H_2_O_2_ solution to continuously treat seeds for 7 days in the dark at room temperature provided the highest germination percentage.

### Validation of the H_2_O_2_ Protocol to Promote Germination

The ability of H_2_O_2_ to promote germination was tested using seeds from 50 randomly selected sugarbeet lines that had been cold stored for 10–37 years. Average germination percentage of these seeds using the H_2_O_2_ treatment reached 73.7% with a range of 38.0–99.0%. This was 49.7% greater than germination observed in water controls (average at 24.0% with a range of 1.0–70.0%). Analysis by *t*-tests indicated that mean percentages of the two treatment were significantly different at probability level of *P* < 0.01 (Table 3), which proved that the H_2_O_2_ protocol significantly enhanced germination of sugarbeet. Correlation analysis found that germination percentages of the 50 lines under the two treatments were highly correlated (*r* = 0.715, *P* < 0.01, Table 3), which means the H_2_O_2_ protocol will have a better chance to improve germination if seeds were able to germinate in water incubation. Particularly, the protocol showed a promising ability of rescuing germplasms such as F1011, F1012 and F1013 that had very low germination percentages using a water incubation (Table 3).

**TABLE 3:** Germination percentage of seeds of 50 sugarbeet lines that were cold stored for 10–37 years after incubation in 1% H_2_O_2_ or water for 8 days.

Line name	Years cold stored	Observed germination (%)	
		H_2_O_2_ treatment	Water control
F1027	10	42.0	6.0
F1032	10	49.0	5.0
FC504cms	11	85.0	8.0
C8747	12	59.0	8.0
F1034	12	67.0	16.0
F1021	13	49.0	15.0
C19	14	64.0	4.0
F1019	16	92.0	18.0
B47	19	97.0	48.0
B50	19	73.0	11.0
F1001	19	77.0	11.0
F1011	19	38.0	1.0
F1013	19	42.0	1.0
F1014	19	72.0	14.0
F1012	20	49.0	2.0
C1451	24	45.0	2.0
C1801	24	94.0	44.0
C191	24	98.0	36.0
F1005	24	67.0	29.0
F1016	24	54.0	13.0
Y318	24	78.0	34.0
Y322	24	95.0	21.0
C211	25	76.0	16.0
C40	25	80.0	33.0
C45	25	61.0	27.0
F1010	25	99.0	60.0
G 241	25	95.0	66.0
GW 359	25	95.0	70.0
C141	26	89.0	25.0
C143	26	66.0	13.0
C1452	26	99.0	30.0
C153	26	86.0	35.0
C156	26	71.0	40.0
C159	26	85.0	23.0
C161	26	80.0	6.0
C172	26	56.0	7.0
C179	26	76.0	11.0
C180	26	98.0	70.0
C1817	26	97.0	34.0
C192	26	74.0	27.0
C22	26	75.0	25.0
C24	26	78.0	33.0
C29	26	95.0	32.0
F1002	30	40.0	17.0
FC712	30	58.0	10.0
PI 467869	30	84.0	22.0
PI 467870	30	98.0	62.0
SP69550	30	64.0	8.0
F1009	33	69.0	23.0
F1006	37	55.0	26.0
Average germination percentage		73.7 ± 18.3	24.0 ± 18.2
Significance through *t*-test		*P* < 0.01	
Correlation of germination between two treatments		*r* = 0.715, *P* < 0.01	

### Germination of Individual Isolated Seeds

When germinating seeds isolated from seedballs, only 4% of F1004 seeds germinated in water incubation for 4 days (Table 4), a germination percentage that was similar to that achieved when incubating F1004 seedballs in water. However, 60% of isolated seeds of F1015 germinated when incubated in water (Table 4), which was much higher than the 0% germination of seedballs incubated in water but close to the germination percentage achieved under H_2_O_2_ treatment. Therefore, difficulties in germinating F1015 seedballs when incubated in water were mainly caused by the physical barrier of the seed coat. In contrast, the consistently low germination rate in F1004 seedballs and isolated seeds indicated that endogenous physiological dormancy was likely to be responsible for inhibiting germination.

**TABLE 4:** Germination of seeds isolated from the seedballs of F1004 and F1015 in distilled water at room temperature under natural light.

Line	Total seeds	Germinated seeds on the day				Total seeds germinated	Germination rate (%)
		1st	2nd	3rd	4th		
F1004	50	0	1	1	0	2	4.0
F1015	50	2	13	15	0	30	60.0

### Germinating Seeds for RNA-seq Analysis

**FIGURE 1:**
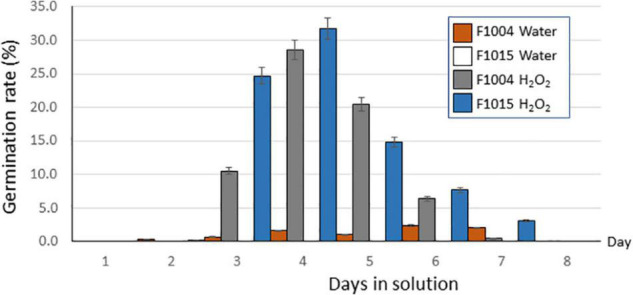
Percent of seeds germinated as a function of time in solution for seedballs of sugarbeet lines F1004 and F1015 that were germinated in a 1% H_2_O_2_ solution or distilled water for 8 days. No seeds of F1015 were germinated under water control.

Of the seedballs used for RNA-seq analysis, germination after 8 days in a H_2_O_2_ solution reached 82.0% in F1015 and 66.4% in F1004, which far exceeded the 0% germination in F1015 and the 7.6% germination in F1004 from water-incubated controls (Figure 1). Germination of seedballs in H_2_O_2_ treatment in both lines occurred predominantly on the 3rd, 4th, 5th, and 6th days, with maximum germination on the 4th day (Figure 1). This agreed with observations made in previous germination tests that seed imbibition started on the second day, germination peaked at the 3rd and 4th days and finished on the 7th and 8th days of the treatment. Therefore, seed samples on the first day of H_2_O_2_ and water treatments were collected as controls for gene expression before germination started, and samples collected on the second day of the treatment represented gene expression after imbibition began in a few seeds under each treatment. Seed samples from the 3rd and 4th days of the treatments, when germination was greatest, were pooled and presumed to have the greatest expression of genes related to germination. Pooled samples from the 5th and 6th days, and the 7th and 8th days of the treatments were collected to assist in identifying genes that diminish in expression during germination.

### RNA-seq Sequencing Summary

RNA extracted from the F1004 seed sample on the second day in the water control was of low quality and was removed from RNA-seq analysis. RNA extracted from the remaining 19 samples that were collected from two sugarbeet lines (F1004 and F1015) under two treatments (H_2_O_2_ treatment vs. water control) on different days (1st, 2nd, pools of 3rd and 4th, 5th and 6th, and 7th and 8th), however, were used to produce RNA sequencing libraries. A total of 86.8 Gb bases of RNA sequence was generated with an average of 4.6 Gb bases per sample. After quality trimming and filtering, 868 million clean reads remained, yielding an average of 45.7 million reads per sample. About 76.7% of the sequences obtained mapped to the sugarbeet RefBeet reference genome with 59.0% of them mapped uniquely to single location (Supplementary Table 1). Overall, a total of 26,668 genes were identified with 22,287 annotated and 4,401 that were novel genes with unknown functions.

### Comparison of Gene Expression Between H_2_O_2_ Treatment and Water Control

**FIGURE 2:**
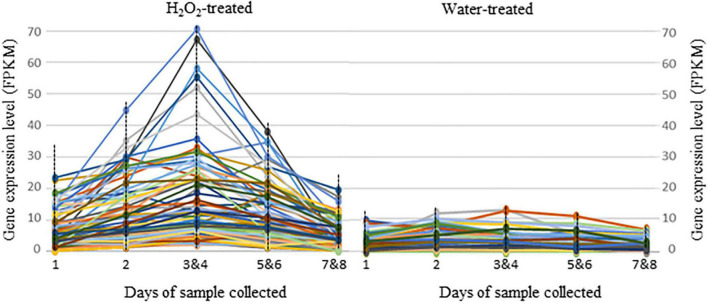
Expression level changes for the 90 DEGs (differentially expressed genes) that were more highly expressed in seeds of sugarbeet lines F1004 and F1015 under H_2_O_2_ treatment. The samples labeled as 1, 2, 3&4, 5&6, and 7&8 correspond to seedball samples collected on the 1st, 2nd, 3rd and 4th, 5th and 6th, and 7th and 8th days of the treatment. Lines with different colors represent different genes. Expression levels and function of genes were shown in Supplementary Table 2.

Comparisons of transcriptome profiles from seed samples collected on different days all showed significant gene expressions difference between H_2_O_2_ treatment and water control in both F1004 and F1015 (Supplementary Figure 1). Combining all expression differences in different samples and genotypes identified a common set of 173 DEGs, of which 90 genes were expressed at higher levels in seeds treated by H_2_O_2_ and 83 genes were expressed at higher levels in seed samples from the water control (Table 5 and Supplementary Table 2). The 90 upregulated DEGs from the H_2_O_2_ treatment mostly were expressed at low levels in samples collected on the first day of the treatments but increased in samples collected on the second day and the pooled samples from the 3rd and 4th days, then declined in pooled samples for the 5th and 6th, and 7th and 8th days of the treatment (Figure 2), which mirrored germination during the 8 days of the experiment (Figure 1). An analysis of gene functions found 62 (68.9%) genes were related to growth, with 23 and 11 genes involved in the cell cycle and DNA replication, respectively, suggesting that H_2_O_2_ treatment promoted gene activities related to cell cycle and growth. Of the remaining H_2_O_2_-promoted DEGs, five were of unknown function, and 12 (13.3%) and 11 (12.2%) were related to the regulation of gene expression and stress responses, respectively. Of the DEGs related to gene expression regulation, nine were transcription regulators, two were involved in signal transduction, and one was involved in a phytohormone-signaling pathway. From the 11 DEGs related to stress responses, seven were involved in responses to oxidative stress, two were involved in defense responses, and two were involved in response to cold and osmotic stress (Table 5 and Supplementary Table 2).

**TABLE 5:** Comparison of the biological functions of genes that were differentially expressed in seedballs germinated in 1% H_2_O_2_ solution (H_2_O_2_ treatment) or distilled water (water control).

Biological function	DEGs with higher expression level^a^	
	H_2_O_2_ treatment	Water control
Expression regulation related		
Transcription regulation	9	3
Phytohormone-signal pathway	1	3
Other signal transduction	2	1
Growth related		
Biosynthesis	3	4
Protein catabolic	2	2
Growth regulation	3	4
Cell cycle and growth	23	7
DNA replication	11	1
Chromosome organization	3	0
Metabolic process	13	10
Translational regulation	2	4
Transporter	2	4
Stress response related		
Defense response	2	3
Oxidative stress response	7	8
Response to cold	1	4
Osmotic stress/water deprivation response	1	11
Cell protection	0	3
Unknown function	5	11
Total	90	83

*^a^DEGs, differentially expressed genes.*

*The complete list of DEGs between H_2_O_2_ treatment and water control and their biological function can be found in Supplementary Table 2.*

**FIGURE 3:**
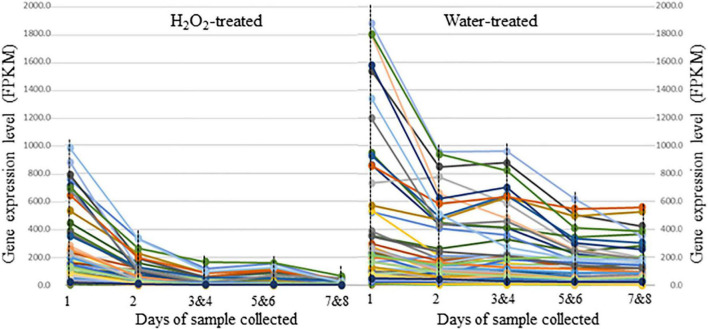
Expression level changes for the 83 DEGs (differentially expressed genes) that were more highly expressed in seeds of sugarbeet lines F1004 and F1015 germinated in water relative to seeds germinated in H_2_O_2_. Samples labeled as 1, 2, 3&4, 5&6, and 7&8 correspond to seedball samples that were collected on the 1st, 2nd, 3rd and 4th, 5th and 6th, and 7th and 8th days. Lines with different colors represented different genes. Expression levels and function of genes were shown in Supplementary Table 2.

Of the 83 DEGs with greater expression in the water controls, 36 (43.4%) were related to growth, 29 (34.9%) were involved in stress responses, and seven (8.4%) were regulators of gene expression. Expression of these DEGs decreased as seeds were incubated for longer durations in either H_2_O_2_ solution or distilled water, but expression declined more rapidly in samples treated with H_2_O_2_ (Figure 3). Of the DEGs with biological functions related to stress responses, eight were involved in response to oxidative stress, four were related to response to cold, 11 were involved in response to osmotic or water deprivation stress, and three corresponded to cell protection. All of these functions suggest that transcripts that were more highly expressed in water-incubated seeds were unlikely to be genes expressed during imbibition since seedballs were not subjected to cold or water deprivation during this time. Rather, these DEGs were presumably stored mRNAs that were produced while seeds were stored under dry and cold conditions or were transcribed when water content declined during seed maturation. Expression of these DEGs progressively decreased during the duration of the experiment in seedballs under both treatments, which is also consistent with a degradation of stored mRNAs during imbibition. Therefore, the faster decline in mRNAs that were likely to be present in stored seedballs under H_2_O_2_ treatment suggests that H_2_O_2_ accelerated the transition of seeds from dormancy to germination.

### Comparison of Gene Expression Between F1004 and F1015 Under H_2_O_2_ Treatment

Germination tests using isolated seeds indicated that germination was restricted by physiological dormancy in F1004 and physical dormancy in F1015. H_2_O_2_ treatment increased germination for both lines, presumably by improving water and oxygen penetration through the pericarp in F1015 and by triggering gene expression changes to overcome physiological dormancy in F1004. Therefore, comparison of gene expression between these two lines when their germination was promoted by H_2_O_2_ could reveal changes in gene expression that are related to the alleviation of physiological dormancy.

Since previous experiments found that incubating seeds in H_2_O_2_ solution for 1 day had no effect on germination and seeds that remained ungerminated after 7 days of H_2_O_2_ treatment were likely to be unviable, the transcription data from seedballs treated with H_2_O_2_ for 2–6 days in the two lines were used to identify DEGs between F1004 and F1015. In total, 370 DEGs were detected between the two lines with 101 and 269 DEGs more highly expressed in F1015 and F1004, respectively (Table 6 and Supplementary Table 3). The significantly higher number of DEGs in F1004 suggests that overcoming physiological dormancy required alteration of a greater number of gene activities than were needed to overcome the physical dormancy of F1015.

**TABLE 6:** Comparison of the biological functions of genes that were differentially expressed in seedballs of sugarbeet lines F1004 and F1015 during germination induced using a 1% H_2_O_2_ solution.

Biological function	DEGs with higher expression level^a^	
	F1015	F1004
Expression regulation related		
Transcription regulation	1	15
ABA-mediated signaling pathway	1	7
GA- mediated signaling pathway	0	1
Auxin-activated signaling pathway	3	0
Other signal transduction	2	5
Growth related		
Biosynthesis	10	4
Protein catabolic	0	1
Growth regulation	4	10
Cell cycle and growth	5	10
DNA replication	1	1
Metabolic process	4	6
Translational modification	4	7
Transporter	10	8
Stress response related		
Defense response	8	3
Oxidative stress response	3	14
Other stress response	7	5
Unknown function	38	172
Total	101	269

*^a^DEGs, differentially expressed genes.*

*The complete list of the DEGs between F1004 and F1015 and their function was shown in Supplementary Table 3.*

Analysis of DEGs by biological function indicated a large difference between the two lines in genes involved in gene expression regulation. For example, F1004 had 28 upregulated DEGs involved in expression regulation of which 15 were transcription regulators, seven were involved in the ABA-mediated signaling pathway, one was involved in the GA-mediated signaling pathway, and five were involved in other signal transduction processes. In contrast, only seven DEGs that were involved in gene expression regulation were expressed at a higher level in F1015, including one transcription regulator, one involved in ABA-mediated signaling, three that were involved in auxin-activated signaling pathways, and two involved in other signal transduction processes (Table 6 and Supplementary Table 3). Overall, this suggests that ABA-mediated signaling pathways, along with a significant number of transcription regulators, played important roles in cell regulation toward overcoming physiological dormancy.

Another significant difference in gene expression between the two lines were the number of DEGs involved in responses to oxidative stress with 14 expressed at higher levels in F1004 but only three that were more highly expressed in F1015. The H_2_O_2_ treatment created an oxidative environment for both lines, but the greater number of genes in line F1004 involved in response to oxidative stress indicates that some oxidative stress response genes may participate in regulating germination rather than merely providing cell protection against oxidative stress. In addition, 38 and 172 DEGs were non-annotated with unknown functions in F1015 and F1004, respectively (Table 6 and Supplementary Table 3). The larger number of unknown DEGs in F1004 provides further evidence that release of physiological dormancy requires more complex changes in gene transcription than release of physical dormancy.

## Discussion

Germination is a key developmental stage in a plant’s life cycle. This research demonstrated that incubating sugarbeet seeds in a 1% H_2_O_2_ solution for 7 days or more at room temperature in the dark maximizes germination percentage. Research also established that H_2_O_2_ treatment can break both physical and physiological dormancies. By using this protocol, germination percentage in 50 sugarbeet lines were significantly increased (Table 3). Acceptable germination was also achieved in germinating an additional 2,000 sugarbeet germplasms through this method (data not shown). Additionally, H_2_O_2_ treatment greatly improved germination of some wheat and sunflower lines that germinated poorly in water, although post-germination root growth of wheat and sunflower was inhibited by H_2_O_2_ (data not shown). Lower H_2_O_2_ concentrations and shorter treatment times reduced the harmful effects of H_2_O_2_ in these species and indicate that H_2_O_2_ concentration and treatment time need to be optimized for each plant species to avoid damage to young seedlings by H_2_O_2_. Fortunately, no harmful effects of H_2_O_2_ were observed when germinating sugarbeet seeds, although seedlings of a few sugarbeet genotypes showed signs of damage if germinated seeds remained in H_2_O_2_ solution beyond 7 days. Thus, timely transplanting the established seedlings into soil is necessary to minimize the toxic effects of H_2_O_2_.

Cold treatment is normally considered helpful for breaking seed dormancy and Yamauchi et al. (2004) found that GA biosynthesis in *Arabidopsis thaliana* seeds was likely stimulated through upregulating the cold-inducible GA biosynthesis genes *GA3ox2* and *GA3ox2* under a low temperature. However, this research indicated that low temperature was not helpful for breaking dormancy of sugarbeet seeds and greatly reduced the effect of H_2_O_2_ in promoting germination. This finding may be due to lower catalase activity at low temperature that reduces H_2_O_2_ catabolism as reported in MacRae and Ferguson (1985). Soaking seeds in water before planting has been recommended by Habib (2010) for enhancing sugarbeet germination in fields, suggesting that an increase in seed hydration may be helpful for germination. However, in our studies, incubation in water for 1–3 days prior to H_2_O_2_ treatment impaired the ability of H_2_O_2_ to promote germination. As the seeds used in this research germinated poorly in water due to their strong dormancy, viability of some seeds may be lost during water pre-incubation due to the extension of the incubation period, which thus reduces germination by delaying H_2_O_2_ treatment. Light is normally considered as a factor that influences germination in many plant species (Shinomura, 1997), and this research also found that darkness increased germination of F1004 seeds in water incubation (Table 2). But for all seeds treated with H_2_O_2_ incubation, no significant effect of darkness in enhancing germination was observed. It is possible that H_2_O_2_ has the overwhelm effect of promoting germination and makes effect of darkness for germination unobservable. Overall, using the H_2_O_2_ protocol from this research for germinating seeds, there was no need to pretreat seeds with low temperature or a water incubation, but darkness is recommended. An additional benefit of using H_2_O_2_ to promote germination is its antimicrobial properties (McDonnell, 2014) that protect germinating seeds from pathogens. Similar protective effects of H_2_O_2_ against pathogens were also made by Schopfer et al. (2001) and Morkunas et al. (2004). Therefore, the protocol using H_2_O_2_ to enhance germination developed here will be useful for rescuing germplasms of sugarbeet or other plant species that have been exposed to pathogens.

Normally, dry quiescent seeds resume metabolic activity during imbibition. However, the seedballs of the two sugarbeet lines used in this research had very low germination percentage when incubated in distilled water. Preincubating seedballs in distilled water greatly reduced germination even when immediately followed by H_2_O_2_ incubation. In contrast, seedballs consistently incubated in a H_2_O_2_ solution had much improved germination, establishing that H_2_O_2_ promotes imbibition and alters gene expression to encourage germination. Comparison of gene transcription profiles of seedballs treated with H_2_O_2_ or water also found that the majority of DEGs that were more highly expressed under H_2_O_2_ treatment were related to growth activities with many involved in cell proliferation, DNA replication and metabolism. In addition, twelve DEGs that were upregulated in H_2_O_2_ treated seedballs participated in gene expression regulation with nine genes encoding transcription regulators and two genes involved in signal transduction processes (Table 5 and Supplementary Table 2). This suggests that exogenous H_2_O_2_ promotes the regulation of gene expression toward germination. However, it is unknown whether endogenous H_2_O_2_ contributes to the expression of genes related to germination under H_2_O_2_ treatment. Further studies, therefore, are needed to separate the external and internal effects of H_2_O_2_ on sugarbeet seed germination to characterize the mechanism by which H_2_O_2_ regulates gene expression and initiates germination.

In contrast, of the 83 DEGs that were more highly expressed in water-treated seedballs, 29 genes were involved in stress responses, with 24 DEGs contributing to responses to cold, osmotic stress, oxidative stress, and water deprivation and four involved in cell protection (Table 5 and Supplementary Table 2). Since no cold, osmotic or water deprivation stress occurred during germination in this study, the DEGs that were more highly expressed in water-treated samples were likely mRNAs synthesized prior to or during cold storage of the seeds. The continuous reduction of these DEGs during H_2_O_2_ or water treatment provides further indication that they were mRNAs stored in seeds. A much sharper reduction of these stored mRNAs in H_2_O_2_ treated seedballs suggests that the faster degradation of these mRNAs improved germination. Therefore, seed dormancy and germination are not only affected by transcriptional regulation during germination, but also by the degradation of mRNAs stored in seeds as has been previously observed in sunflower by El-Maarouf-Bouteau et al. (2015).

The faster degradation of mRNAs in H_2_O_2_ treated seedballs may be largely due to oxidative damage. Under the oxidative conditions created by incubating in a H_2_O_2_ solution, oxidative damage to mRNA can occur due to mRNA cellular localization, its single-stranded structure, and a lack of mRNA repair mechanisms in cells (Kong and Lin, 2010). Oxidative damage to stored mRNA can inhibit protein synthesis and protein degradation, thereby interrupting the processes that maintain dormancy as well as acting as a positive signal to initiate processes related to germination (El-Maarouf-Bouteau et al., 2013; Chmielowska-Bak et al., 2015). However, we found the level of stored mRNAs was greatly reduced in seeds on the first day of H_2_O_2_ treatment relative to the corresponding sample in the water control (Figure 3), even though germination was not improved for seeds incubated in H_2_O_2_ for only 1 day. This indicates that the degradation of mRNA alone is not sufficient to overcome seed dormancy.

Germination tests using individual sugarbeet seeds isolated from the woody seedballs found that poor germination under water treatment in F1015 was primarily due to the physical dormancy imposed by the dense inner layer of pericarp, whereas the low germination of F1004 was mostly caused by physiological dormancy of the embryo that inhibited germination. This research established that H_2_O_2_ breaks both physical and physiological dormancies. Overcoming physical dormancy with H_2_O_2_ and ROS has been demonstrated in lettuce since exogenous ROS increase the percentage of endosperm caps that ruptured during germination (Zhang et al., 2014). Oxidative reactions involving proteins and other intercellular substances are believed to loosen cell walls (Wojtyla et al., 2016) and promote water and oxygen penetration through the mechanic barriers of the seed. Mechanisms by which H_2_O_2_ treatment breaks physiological dormancy, however, are largely unknown.

A comparison of transcripts in H_2_O_2_-treated seed samples of F1004 and F1015 provided insight into genes that may be involved in overcoming physiological dormancy. F1004 had more upregulated DEGs than F1015 which suggests that alleviating physiological dormancy involves more gene activities and biological processes than alleviating physical dormancy (Table 6 and Supplementary Table 3). Of all DEGs between the two lines, F1004 had 28 DEGs involved in transcription regulation, ABA-mediated signaling pathways, GA-mediated signaling pathways and other signal transduction processes, whereas F1015 only had seven upregulated DEGs related to gene expression regulation. More DEGs involved in ABA or GA-mediated signaling pathways in F1004 indicates that the well-known action between ABA and GA plays a role in releasing physiological dormancy and initiating germination. The greater prevalence of DEGs related to transcription regulators in F1004 also indicate that H_2_O_2_ was involved in gene expression regulatory changes that assisted in breaking physiological dormancy. Several studies have found that H_2_O_2_ directly acts as a signaling molecule and participates in crosstalk with phytohormones to regulate germination (Barba-Espín et al., 2010; El-Maarouf-Bouteau et al., 2015). Therefore, H_2_O_2_ interactions with phytohormones likely have a role in regulating gene expression to promote the transition of F1004 seeds from dormancy to germination.

Another notable difference between F1004 and F1015 was the number of DEGs involved in oxidative stress responses. Of these DEGs, 14 were highly expressed in F1004 and only three were more highly expressed in F1015. Since H_2_O_2_ treatment released physical dormancy of F1015 seeds, gene expression in H_2_O_2_-treated F1015 seeds was expected to be similar to gene activities that occurred in seeds without dormancy during germination. The three upregulated DEGs in F1015 that were involved in responding to oxidative stress, therefore, were likely to contribute to cell protection against the oxidative conditions of H_2_O_2_ treatment. In contrast, the significantly greater number of DEGs for oxidative responses in F1004 may contribute to regulating oxidation levels in seeds to allow them to maintain the “oxidative window” that is critical for germination as proposed by Bailly et al. (2008) in addition to their role in cell protection responses. In addition, there were 172 unannotated DEGs that were more highly expressed in F1004. Annotation of these genes would be potentially helpful to further understanding mechanisms by which sugarbeet seeds overcome physiological dormancy.

## Conclusion

This research developed an effective protocol to enhance sugarbeet germination by continuously incubating seedballs in a 1% H_2_O_2_ solution in the dark for 7 days. This method provides a useful method for rescuing plant germplasms with poor germination. Gene expression analysis indicated that H_2_O_2_ treatment not only promoted gene activities related to cell proliferation and growth, but also led to faster degradation of mRNAs stored in seeds to promote the transition from seed dormancy to germination. Comparison of transcripts during germination in seeds of F1004 and F1015 with physiological and physical dormancies, respectively, found a greater number of DEGs were involved in overcoming physiological dormancy of F1004, with many DEGs related to regulating gene expression and responses to oxidative stress. This finding suggests that regulating gene expression along with adjusting the oxidative level in seeds played key roles in breaking the physiological dormancy of this sugarbeet line.

## Data Availability Statement

The datasets presented in this study can be found in online repositories. The names of the repository/repositories and accession number(s) can be found below: National Center for Biotechnology Information (NCBI) BioProject database under accession number PRJNA787080 (https://www.ncbi.nlm.nih.gov/bioproject/PRJNA787080/).

## Author Contributions

CC designed the project, conducted bench work and data analysis, and prepared the manuscript. RP conducted bench work. MB involved project design and reviewed the manuscript. KF helped on data analysis and revised the manuscript. All authors contributed to the article and approved the submitted version.

## Conflict of Interest

The authors declare that the research was conducted in the absence of any commercial or financial relationships that could be construed as a potential conflict of interest.

## Publisher’s Note

All claims expressed in this article are solely those of the authors and do not necessarily represent those of their affiliated organizations, or those of the publisher, the editors and the reviewers. Any product that may be evaluated in this article, or claim that may be made by its manufacturer, is not guaranteed or endorsed by the publisher.

## Footnotes

1
https://www.rdocumentation.org/packages/stats/versions/3.6.2/topics/aov

2
https://www.rdocumentation.org/packages/agricolae/versions/1.3-5/topics/LSD.test

3
https://github.com/BGI-flexlab/SOAPnuke

4
https://www.ncbi.nlm.nih.gov/genome/?term=Beta%20Vulgaris

5
https://stat.ethz.ch/R-manual/R-devel/library/stats/html/Hypergeometric.html

6
https://www.ncbi.nlm.nih.gov/

## Supplementary Material

Supplementary Figure 1 Plots of transcriptome comparisons through Poisson Distribution method between the paired treatments of incubating seedballs of F1004 and F1015 in H_2_O_2_ solution and water. For each of plots, *X*-axis represents the value of log2 transformed mean expression level, *Y*-axis represents value of log2 transformed expression level fold change calculated from dividing level of H_2_O_2_ treatment with that in water control. Red dots represent up-regulated DEGs in H_2_O_2_ treatment, blue dots represent down-regulated ones, and gray points represent non-DEGs.
