# Supplementary material for: Mechanism of Sugarbeet Seed Germination Enhanced by Hydrogen Peroxide
Source: Front Plant Sci. 2022 Apr 25;13:888519. doi: 10.3389/fpls.2022.888519 (PMC9082935; doi:10.3389/fpls.2022.888519)
Supplement: Supplementary file 1 [file Table_2.DOCX]

**Supplementary Table S1.** Statistics describing sequencing and mapping results of RNA isolated from seedballs of sugarbeet lines F1004 and F1015 that were germinated in H_2_O_2_ or distilled water for 8 days^a^

| **Sample^b^** | **Raw Reads (Million)** | **Clean Reads (Million)** | **Mapped to RefBeet (%)** | **Uniquely Mapped (%)** |
| --- | --- | --- | --- | --- |
| F1004P1 | 45.6 | 42.7 | 70.2 | 53.8 |
| F1004P2 | 50.0 | 47.1 | 80.6 | 63.0 |
| F1004P34 | 50.0 | 47.0 | 85.7 | 66.9 |
| F1004P56 | 50.0 | 46.8 | 86.1 | 66.9 |
| F1004P78 | 50.0 | 47.1 | 76.1 | 59.0 |
| F1004W1 | 50.0 | 46.9 | 82.6 | 63.8 |
| F1004W34 | 50.0 | 47.0 | 78.4 | 61.1 |
| F1004W56 | 50.0 | 46.8 | 79.1 | 61.8 |
| F1004W78 | 50.0 | 47.0 | 78.7 | 61.3 |
| F1015P1 | 50.0 | 46.9 | 86.7 | 66.1 |
| F1015P2 | 50.0 | 46.8 | 87.1 | 65.9 |
| F1015P34 | 50.0 | 46.9 | 87.5 | 66.7 |
| F1015P56 | 50.0 | 47.0 | 86.1 | 66.0 |
| F1015P78 | 34.2 | 32.0 | 49.9 | 37.6 |
| F1015W1 | 45.9 | 43.1 | 69.3 | 52.3 |
| F1015W2 | 50.0 | 47.0 | 81.8 | 62.4 |
| F1015W34 | 50.0 | 46.8 | 67.6 | 51.4 |
| F1015W56 | 50.0 | 46.6 | 55.5 | 42.7 |
| F1015W78 | 50.0 | 46.7 | 68.1 | 51.8 |

^a^The RefBeet reference genome (Dohm et al. 2014) is available from <https://www.ncbi.nlm.nih.gov/genome/?term=Beta%20Vulgaris>.

^b^For sample identifiers, first four characters identify the sugarbeet line (F1004 or F1015). P or W denote germination treatment (H_2_O_2_ [P] or water [W]). Final numbers identify the day on which seed samples were collected.
