# Supplementary material for: Mechanism of Sugarbeet Seed Germination Enhanced by Hydrogen Peroxide
Source: Front Plant Sci. 2022 Apr 25;13:888519. doi: 10.3389/fpls.2022.888519 (PMC9082935; doi:10.3389/fpls.2022.888519)
Supplement: Supplementary file 3 [file Table_4.DOCX]

**Supplementary Table S3.** List of gene product and biological function of differentially expressed genes (DEGs) between sugarbeet lines F1004 and F1015 during germination under H_2_O_2_ treatment

| **Gene ID** | **Gene product** | **Biological function** | |
| --- | --- | --- | --- |
| Genes expressed at a higher level in F1005: | | |  |
| 104894178 | L10-interacting MYB domain-containing protein-like | Transcription regulation | |
| 104894843 | protein S-acyltransferase 18 | Signal transduction | |
| 104904624 | root phototropism protein 3 | Signal transduction | |
| 104897434 | MLP-like protein 423 | ABA-activated signaling pathway | |
| 104885257 | probable glutathione S-transferase parC | auxin-activated signaling pathway | |
| 104898547 | auxin-binding protein ABP19a-like | auxin-activated signaling pathway | |
| 104884870 | auxin-responsive protein IAA32 | auxin-activated signaling pathway | |
| 104892093 | 7-deoxyloganetin glucosyltransferase | Biosynthesis | |
| 104900994 | ATP synthase gamma chain, chloroplastic | Biosynthesis | |
| 104893901 | acyl carrier protein 1, chloroplastic | Biosynthesis | |
| 104888150 | caffeic acid 3-O-methyltransferase 2 | Biosynthesis | |
| 104901928 | codeine O-demethylase-like | Biosynthesis | |
| 104892645 | granule-bound starch synthase 1, chloroplastic/amyloplastic | Biosynthesis | |
| 104908747 | beta-amyrin synthase | Biosynthesis | |
| 104903946 | photosystem I reaction center subunit III, chloroplastic | Biosynthesis | |
| 104902012 | thylakoid lumenal 16.5 kDa protein, chloroplastic | Biosynthesis | |
| 104898134 | serine/threonine-protein kinase STN8, chloroplastic | Biosynthesis | |
| 104902014 | axial regulator YABBY 5 | Growth regulation | |
| 104901971 | BEL1-like homeodomain protein 9 | Growth regulation | |
| 104907055 | GPI-anchored protein LLG1-like | Growth regulation | |
| 104896351 | piriformospora indica-insensitive protein 2 | Growth regulation | |
| 104892924 | transcriptional regulator SUPERMAN | Cell cycle and growth | |
| 104891868 | exopolygalacturonase clone GBGA483 | Cell cycle and growth | |
| 104901977 | 36.4 kDa proline-rich protein | Cell cycle and growth | |
| 109135582 | peptidyl-prolyl cis-trans isomerase FKBP16-4, chloroplastic-like | Cell cycle and growth | |
| 104886553 | LRR receptor-like serine/threonine-protein kinase ERECTA | Cell cycle and growth | |
| 104895012 | Werner Syndrome-like exonuclease | DNA replication | |
| 104903549 | 21 kDa seed protein | Metabolic process | |
| 104895151 | ras-related protein RAB1BV-like | Metabolic process | |
| 104885084 | D-amino-acid transaminase, chloroplastic | Metabolic process | |
| 104888815 | cytosolic sulfotransferase 5 | Metabolic process | |
| 104890740 | putative pentatricopeptide repeat-containing protein At1g12700, mitochondrial | Translation modification | |
| 104901135 | putative receptor-like protein kinase | Translation modification | |
| 104904403 | putative receptor-like protein kinase | Translation modification | |
| Novel_G002359 | 40S ribosomal protein S2-2 | Translation modification | |
| 104908210 | bidirectional sugar transporter SWEET4-like | Transporter | |
| 104906057 | cyclic nucleotide-gated ion channel 17 | Transporter | |
| 104905911 | basic blue protein | Transporter | |
| 104897913 | protein NRT1/ PTR FAMILY 5.10-like | Transporter | |
| 104894068 | cation/H(+) antiporter 14-like | Transporter | |
| 104908800 | putative ER lumen protein-retaining receptor C28H8.4 | Transporter | |
| 109133994 | eukaryotic translation initiation factor 2 subunit beta-like | Transporter | |
| 104900626 | triose phosphate/phosphate translocator, chloroplastic | Transporter | |
| 104896649 | amino-acid permease BAT1 homolog | Transporter | |
| Novel_G002637 | AP-4 complex subunit mu isoform X1 | Transporter | |
| 104883955 | MLP-like protein 43 | Defense response | |
| 104898166 | probable disease resistance protein | Defense response | |
| 104906068 | L-ornithine N5-acetyltransferase NATA1 | Defense response | |
| 104896995 | putative calcium-transporting ATPase 11, plasma membrane-type | Defense response | |
| 104906081 | ribulose bisphosphate carboxylase small chain | Defense response | |
| 104889235 | polygalacturonase inhibitor | Defense response | |
| 104892935 | protein LOL1 | Defense response | |
| 104903991 | snakin-2 | Defense response | |
| 104897405 | aldehyde dehydrogenase family 2 member B7, mitochondrial | Oxidative stress response | |
| 104905530 | photosystem II 22 kDa protein, chloroplastic | Oxidative stress response | |
| 104900592 | peroxidase 19 | Oxidative stress response | |
| 104888293 | transcription factor SPEECHLESS | Other stress response | |
| 104893018 | rhodanese-like domain-containing protein 10 | Other stress response | |
| 104896210 | light-harvesting complex-like protein OHP2, chloroplastic | Other stress response | |
| 104892849 | chlorophyll a-b binding protein CP29.3, chloroplastic | Other stress response | |
| 104903711 | ferredoxin-1, chloroplastic-like | Other stress response | |
| 104898096 | UDP-glycosyltransferase 79B6 | Other stress response | |
| 104900220 | UDP-glycosyltransferase 87A1-like | Other stress response | |
| Novel_G002676 | inactive protein kinase SELMODRAFT_444075-like isoform X1 | Unknown | |
| 104885243 | F-box/FBD/LRR-repeat protein | Unknown | |
| 104892060 | thylakoid lumenal protein TL20.3, chloroplastic | Unknown | |
| 104889873 | hypothetical protein | Unknown | |
| 104894025 | hypothetical protein | Unknown | |
| 104895284 | hypothetical protein | Unknown | |
| 104896644 | hypothetical protein | Unknown | |
| Novel_G002925 | hypothetical protein | Unknown | |
| Novel_G003163 | hypothetical protein | Unknown | |
| Novel_G003665 | hypothetical protein | Unknown | |
| 104886236 | uncharacterized protein | Unknown | |
| 104889405 | uncharacterized protein | Unknown | |
| 104892071 | uncharacterized protein | Unknown | |
| 104894111 | uncharacterized protein | Unknown | |
| 104902088 | uncharacterized protein | Unknown | |
| 104902949 | uncharacterized protein | Unknown | |
| 104906334 | uncharacterized protein | Unknown | |
| 104906761 | uncharacterized protein | Unknown | |
| 104907276 | uncharacterized protein | Unknown | |
| 104907406 | uncharacterized protein | Unknown | |
| 104907578 | uncharacterized protein | Unknown | |
| 104908103 | uncharacterized protein | Unknown | |
| 109134538 | uncharacterized protein | Unknown | |
| 109134882 | uncharacterized protein | Unknown | |
| 109136335 | uncharacterized protein | Unknown | |
| Novel_G000278 | uncharacterized protein | Unknown | |
| Novel_G000381 | uncharacterized protein | Unknown | |
| Novel_G001318 | uncharacterized protein | Unknown | |
| Novel_G001615 | uncharacterized protein | Unknown | |
| Novel_G001667 | uncharacterized protein | Unknown | |
| Novel_G001911 | uncharacterized protein | Unknown | |
| Novel_G002156 | uncharacterized protein | Unknown | |
| Novel_G002544 | uncharacterized protein | Unknown | |
| Novel_G003397 | uncharacterized protein | Unknown | |
| Novel_G003666 | uncharacterized protein | Unknown | |
| Novel_G004177 | uncharacterized protein | Unknown | |
| Novel_G004260 | uncharacterized protein | Unknown | |
| Novel_G004288 | uncharacterized protein | Unknown | |
| Genes expressed at a higher level in F1004: | | |  |
| 104887908 | transcription factor UNE10-like | Transcription regulation | |
| 104883246 | LOB domain-containing protein 1 | Transcription regulation | |
| 104886638 | transcription factor DIVARICATA-like | Transcription regulation | |
| 104899922 | TATA-box-binding protein 2-like | Transcription regulation | |
| Novel_G003210 | splicing factor U2af small subunit B | Transcription regulation | |
| Novel_G002898 | protein FAR-RED ELONGATED HYPOCOTYL 3-like | Transcription regulation | |
| 104885603 | ankyrin repeat-containing protein At5g02620 | Transcription regulation | |
| 104891602 | NAC domain-containing protein 72 | Transcription regulation | |
| 104891774 | probable WRKY transcription factor 46 | Transcription regulation | |
| 104890265 | protein FAR1-RELATED SEQUENCE 5-like | Transcription regulation | |
| Novel_G001727 | probable cyclin-dependent serine/threonine-protein kinase DDB_G0292550 | Transcription regulation | |
| Novel_G003864 | protein FAR1-RELATED SEQUENCE 5-like | Transcription regulation | |
| Novel_G000893 | protein FAR1-RELATED SEQUENCE 5-like | Transcription regulation | |
| 104888873 | L10-interacting MYB domain-containing protein-like | Transcription regulation | |
| Novel_G003023 | probable protein arginine N-methyltransferase 3 | Transcription regulation | |
| 104888465 | calmodulin | Signal transduction | |
| Novel_G000440 | probable inactive receptor-like protein kinase | Signal transduction | |
| 104884599 | G-type lectin S-receptor-like serine/threonine-protein kinase | Signal transduction | |
| 104902767 | E3 ubiquitin-protein ligase ZSWIM2-like | Signal transduction | |
| 104899774 | ankyrin-1 | Signal transduction | |
| Novel_G001091 | RNA polymerase II C-terminal domain phosphatase-like 4 | ABA-activated signaling pathway | |
| 104896121 | ethylene-responsive transcription factor ABR1 | ABA-activated signaling pathway | |
| 104903353 | ethylene-responsive transcription factor ERF054 | ABA-activated signaling pathway | |
| 104883808 | O-acyltransferase WSD1-like | ABA-activated signaling pathway | |
| Novel_G001784 | dynamin-2B | ABA-activated signaling pathway | |
| 104892899 | em-like protein GEA6 | ABA-activated signaling pathway | |
| 104897483 | GEM-like protein 5 | ABA-activated signaling pathway | |
| 104889429 | probable F-box protein At5g04010 | GA-activated signaling pathway | |
| 104892042 | 7-deoxyloganetin glucosyltransferase | Biosynthesis | |
| 104895655 | 7-deoxyloganetin glucosyltransferase-like | Biosynthesis | |
| 104900204 | folic acid synthesis protein FOL1 | Biosynthesis | |
| 104908451 | UDP-glycosyltransferase 74B1 | Biosynthesis | |
| 104908937 | polyubiquitin 4 | Protein catabolic | |
| Novel_G001374 | phosphatidate cytidylyltransferase 1-like | Growth regulation | |
| Novel_G003596 | protein OBERON 3 | Growth regulation | |
| 104906491 | subtilisin-like protease SBT5.4 | Growth regulation | |
| Novel_G001294 | zinc finger BED domain-containing protein RICESLEEPER 2-like | Growth regulation | |
| Novel_G000695 | zinc finger BED domain-containing protein RICESLEEPER 2-like | Growth regulation | |
| 104893683 | zinc finger BED domain-containing protein RICESLEEPER 4-like | Growth regulation | |
| Novel_G001132 | auxin transport protein BIG | Growth regulation | |
| Novel_G001133 | auxin transport protein BIG | Growth regulation | |
| 104902116 | probable strigolactone esterase DAD2 | Growth regulation | |
| Novel_G003598 | lysophospholipid acyltransferase LPEAT1 isoform X1 | Growth regulation | |
| 104906195 | fimbrin-1 | Cell cycle and growth | |
| Novel_G004290 | DNA ligase 1-like | Cell cycle and growth | |
| 104889587 | glycosyltransferase-like | Cell cycle and growth | |
| 104905250 | fasciclin-like arabinogalactan protein 21 | Cell cycle and growth | |
| 104893214 | tetraspanin-7 | Cell cycle and growth | |
| Novel_G002673 | pherophorin-C2 protein precursor | Cell cycle and growth | |
| 104889293 | shikimate O-hydroxycinnamoyltransferase | Cell cycle and growth | |
| Novel_G001774 | kinesin-like protein KIN-7O isoform X2 | Cell cycle and growth | |
| Novel_G004348 | protein SIEVE ELEMENT OCCLUSION B | Cell cycle and growth | |
| Novel_G000560 | zinc finger MYM-type protein 1-like | Cell cycle and growth | |
| 104888029 | histone H3.3-like | DNA replication | |
| 104899807 | fructan 6-exohydrolase | Metabolic process | |
| 104884234 | putative beta-galactosidase | Metabolic process | |
| 104896067 | glucan endo-1,3-beta-glucosidase 8 | Metabolic process | |
| Novel_G001023 | serine decarboxylase-like | Metabolic process | |
| 104901166 | NEDD8-specific protease 1 | Metabolic process | |
| 104883553 | aspartic proteinase PCS1 | Metabolic process | |
| Novel_G004106 | putative pentatricopeptide repeat-containing protein At5g06400, mitochondrial | Translation modification | |
| 104892435 | dolichyl-diphosphooligosaccharide--protein glycosyltransferase subunit STT3A-like | Translation modification | |
| 104890556 | DEAD-box ATP-dependent RNA helicase 10 | Translation modification | |
| 104886480 | protein gar2-like | Translation modification | |
| Novel_G000806 | WD repeat-containing protein 55 | Translation modification | |
| 104891686 | protein translation factor SUI1 homolog | Translation modification | |
| Novel_G002782 | probable tRNA (guanine(26)-N(2))-dimethyltransferase 1 isoform X1 | Translation modification | |
| 109134622 | acyl-CoA-binding domain-containing protein 6-like | Transporter | |
| Novel_G001794 | kinesin-like protein KIN-UB | Transporter | |
| 104902662 | organic cation/carnitine transporter 2 | Transporter | |
| 104899852 | aluminum-activated malate transporter 2-like | Transporter | |
| 104906455 | potassium channel KAT3 | Transporter | |
| 104888527 | short-chain dehydrogenase TIC 32, chloroplastic | Transporter | |
| 104897250 | ABC transporter B family member 9 | Transporter | |
| Novel_G003899 | SPX domain-containing membrane protein | Transporter | |
| 104905533 | putative disease resistance protein RGA3 | Defense response | |
| Novel_G002351 | wall-associated receptor kinase-like 6 isoform X1 | Defense response | |
| 104900433 | mitogen-activated protein kinase kinase kinase A-like | Defense response | |
| 104903827 | isocitrate dehydrogenase [NAD] regulatory subunit 1, mitochondrial | Oxidative stress response | |
| 104889700 | probable aldo-keto reductase 5 | Oxidative stress response | |
| 104900052 | tropinone reductase homolog At2g29290-like | Oxidative stress response | |
| 104887890 | cytochrome P450 71B9-like | Oxidative stress response | |
| 104904822 | cytochrome P450 76AD1-like | Oxidative stress response | |
| 104894235 | cytochrome P450 CYP72A219 | Oxidative stress response | |
| 104890675 | galactinol synthase 2 | Oxidative stress response | |
| 104896580 | keratin, type II cytoskeletal I-like | Oxidative stress response | |
| 104903543 | nematode resistance protein-like HSPRO2 | Oxidative stress response | |
| 104894931 | peroxidase 50 | Oxidative stress response | |
| 104908267 | peroxidase P7 | Oxidative stress response | |
| 104900166 | zinc finger protein ZAT12 | Oxidative stress response | |
| 104884047 | serine/threonine-protein kinase OXI1 | Oxidative stress response | |
| 104894821 | galactinol synthase 1 | Oxidative stress response | |
| 104883494 | stress protein DDR48-like | Other stress response | |
| Novel_G000239 | proline-rich receptor-like protein kinase PERK5 | Other stress response | |
| 104895803 | probable nucleoredoxin 1 | Other stress response | |
| 104895915 | NAC domain-containing protein 19 | Other stress response | |
| 104901718 | heat shock 70 kDa protein-like | Other stress response | |
| Novel_G000441 | hypothetical protein | Unknown | |
| Novel_G002047 | hypothetical protein | Unknown | |
| Novel_G003816 | hypothetical protein | Unknown | |
| 104883866 | hypothetical protein | Unknown | |
| 104888366 | hypothetical protein | Unknown | |
| Novel_G003423 | hypothetical protein | Unknown | |
| Novel_G003908 | hypothetical protein | Unknown | |
| Novel_G000982 | hypothetical protein | Unknown | |
| Novel_G002668 | hypothetical protein | Unknown | |
| 104898801 | uncharacterized protein | Unknown | |
| 104882998 | uncharacterized protein | Unknown | |
| 104884521 | uncharacterized protein | Unknown | |
| 104884553 | uncharacterized protein | Unknown | |
| 104891492 | uncharacterized protein | Unknown | |
| 104891761 | uncharacterized protein | Unknown | |
| 104891824 | uncharacterized protein | Unknown | |
| 104893396 | uncharacterized protein | Unknown | |
| 104893440 | uncharacterized protein | Unknown | |
| 104894204 | uncharacterized protein | Unknown | |
| 104894275 | uncharacterized protein | Unknown | |
| 104894423 | uncharacterized protein | Unknown | |
| 104894547 | uncharacterized protein | Unknown | |
| 104895834 | uncharacterized protein | Unknown | |
| 104896865 | uncharacterized protein | Unknown | |
| 104896958 | uncharacterized protein | Unknown | |
| 104898244 | uncharacterized protein | Unknown | |
| 104898349 | uncharacterized protein | Unknown | |
| 104898930 | uncharacterized protein | Unknown | |
| 104900388 | uncharacterized protein | Unknown | |
| 104903079 | uncharacterized protein | Unknown | |
| 104905825 | uncharacterized protein | Unknown | |
| 104906603 | uncharacterized protein | Unknown | |
| Novel_G003828 | uncharacterized protein | Unknown | |
| Novel_G002360 | uncharacterized protein | Unknown | |
| Novel_G001033 | uncharacterized protein | Unknown | |
| Novel_G002103 | uncharacterized protein | Unknown | |
| Novel_G001704 | uncharacterized protein | Unknown | |
| Novel_G000115 | uncharacterized protein | Unknown | |
| Novel_G003259 | uncharacterized protein | Unknown | |
| Novel_G001805 | uncharacterized protein | Unknown | |
| Novel_G000465 | uncharacterized protein | Unknown | |
| Novel_G003010 | uncharacterized protein | Unknown | |
| Novel_G003815 | uncharacterized protein | Unknown | |
| Novel_G004318 | uncharacterized protein | Unknown | |
| Novel_G003915 | uncharacterized protein | Unknown | |
| Novel_G002033 | uncharacterized protein | Unknown | |
| Novel_G003443 | uncharacterized protein | Unknown | |
| Novel_G000004 | uncharacterized protein | Unknown | |
| Novel_G002296 | uncharacterized protein | Unknown | |
| Novel_G003255 | uncharacterized protein | Unknown | |
| Novel_G000174 | uncharacterized protein | Unknown | |
| Novel_G003701 | uncharacterized protein | Unknown | |
| Novel_G003528 | uncharacterized protein | Unknown | |
| Novel_G000992 | uncharacterized protein | Unknown | |
| Novel_G003585 | uncharacterized protein | Unknown | |
| Novel_G001506 | uncharacterized protein | Unknown | |
| Novel_G002011 | uncharacterized protein | Unknown | |
| Novel_G002496 | uncharacterized protein | Unknown | |
| Novel_G003799 | uncharacterized protein | Unknown | |
| Novel_G004204 | uncharacterized protein | Unknown | |
| Novel_G002001 | uncharacterized protein | Unknown | |
| Novel_G002151 | uncharacterized protein | Unknown | |
| Novel_G001482 | uncharacterized protein | Unknown | |
| Novel_G003289 | uncharacterized protein | Unknown | |
| Novel_G001519 | uncharacterized protein | Unknown | |
| Novel_G001857 | uncharacterized protein | Unknown | |
| Novel_G002212 | uncharacterized protein | Unknown | |
| Novel_G001660 | uncharacterized protein | Unknown | |
| Novel_G003963 | uncharacterized protein | Unknown | |
| Novel_G001813 | uncharacterized protein | Unknown | |
| Novel_G004112 | uncharacterized protein | Unknown | |
| Novel_G004113 | uncharacterized protein | Unknown | |
| Novel_G004165 | uncharacterized protein | Unknown | |
| Novel_G000974 | uncharacterized protein | Unknown | |
| Novel_G000890 | uncharacterized protein | Unknown | |
| Novel_G002729 | uncharacterized protein | Unknown | |
| Novel_G003300 | uncharacterized protein | Unknown | |
| Novel_G003989 | uncharacterized protein | Unknown | |
| Novel_G003725 | uncharacterized protein | Unknown | |
| Novel_G003608 | uncharacterized protein | Unknown | |
| Novel_G004107 | uncharacterized protein | Unknown | |
| Novel_G003071 | uncharacterized protein | Unknown | |
| Novel_G004155 | uncharacterized protein | Unknown | |
| Novel_G004006 | uncharacterized protein | Unknown | |
| Novel_G002727 | uncharacterized protein | Unknown | |
| Novel_G002750 | uncharacterized protein | Unknown | |
| Novel_G004352 | uncharacterized protein | Unknown | |
| Novel_G002025 | uncharacterized protein | Unknown | |
| Novel_G001720 | uncharacterized protein | Unknown | |
| Novel_G001917 | uncharacterized protein | Unknown | |
| Novel_G001924 | uncharacterized protein | Unknown | |
| Novel_G003307 | uncharacterized protein | Unknown | |
| Novel_G001881 | uncharacterized protein | Unknown | |
| Novel_G001504 | uncharacterized protein | Unknown | |
| Novel_G003322 | uncharacterized protein | Unknown | |
| Novel_G000898 | uncharacterized protein | Unknown | |
| Novel_G000264 | uncharacterized protein | Unknown | |
| Novel_G002646 | uncharacterized protein | Unknown | |
| Novel_G002357 | uncharacterized protein | Unknown | |
| Novel_G003913 | uncharacterized protein | Unknown | |
| Novel_G000807 | uncharacterized protein | Unknown | |
| Novel_G003923 | uncharacterized protein | Unknown | |
| Novel_G002161 | uncharacterized protein | Unknown | |
| Novel_G002834 | uncharacterized protein | Unknown | |
| Novel_G002488 | uncharacterized protein | Unknown | |
| Novel_G001648 | uncharacterized protein | Unknown | |
| Novel_G003468 | uncharacterized protein | Unknown | |
| Novel_G000489 | uncharacterized protein | Unknown | |
| Novel_G000384 | uncharacterized protein | Unknown | |
| Novel_G004230 | uncharacterized protein | Unknown | |
| Novel_G003118 | uncharacterized protein | Unknown | |
| Novel_G001779 | uncharacterized protein | Unknown | |
| Novel_G002287 | uncharacterized protein | Unknown | |
| Novel_G001714 | uncharacterized protein | Unknown | |
| Novel_G000678 | uncharacterized protein | Unknown | |
| Novel_G000871 | uncharacterized protein | Unknown | |
| Novel_G001990 | uncharacterized protein | Unknown | |
| Novel_G000885 | uncharacterized protein | Unknown | |
| Novel_G002242 | uncharacterized protein | Unknown | |
| Novel_G003004 | uncharacterized protein | Unknown | |
| Novel_G003188 | uncharacterized protein | Unknown | |
| Novel_G004400 | uncharacterized protein | Unknown | |
| Novel_G002348 | uncharacterized protein | Unknown | |
| Novel_G000155 | uncharacterized protein | Unknown | |
| Novel_G002081 | uncharacterized protein | Unknown | |
| Novel_G002223 | uncharacterized protein | Unknown | |
| Novel_G002533 | uncharacterized protein | Unknown | |
| Novel_G002462 | uncharacterized protein | Unknown | |
| Novel_G003589 | uncharacterized protein | Unknown | |
| Novel_G001131 | uncharacterized protein | Unknown | |
| Novel_G002484 | uncharacterized protein | Unknown | |
| Novel_G003421 | uncharacterized protein | Unknown | |
| Novel_G002196 | uncharacterized protein | Unknown | |
| Novel_G000839 | uncharacterized protein | Unknown | |
| Novel_G000918 | uncharacterized protein | Unknown | |
| Novel_G002641 | uncharacterized protein | Unknown | |
| Novel_G003738 | uncharacterized protein | Unknown | |
| Novel_G001363 | uncharacterized protein | Unknown | |
| Novel_G001606 | uncharacterized protein | Unknown | |
| Novel_G003005 | uncharacterized protein | Unknown | |
| Novel_G004353 | uncharacterized protein | Unknown | |
| Novel_G000913 | uncharacterized protein | Unknown | |
| Novel_G001943 | uncharacterized protein | Unknown | |
| Novel_G002249 | uncharacterized protein | Unknown | |
| Novel_G000978 | uncharacterized protein | Unknown | |
| Novel_G003804 | uncharacterized protein | Unknown | |
| Novel_G002977 | uncharacterized protein | Unknown | |
| Novel_G002298 | uncharacterized protein | Unknown | |
| Novel_G003761 | uncharacterized protein | Unknown | |
| Novel_G003180 | uncharacterized protein | Unknown | |
| Novel_G000713 | uncharacterized protein | Unknown | |
| Novel_G001856 | uncharacterized protein | Unknown | |
| Novel_G000705 | uncharacterized protein | Unknown | |
| Novel_G001664 | uncharacterized protein | Unknown | |
| Novel_G001681 | uncharacterized protein | Unknown | |
| Novel_G001590 | uncharacterized protein | Unknown | |
| Novel_G003487 | uncharacterized protein | Unknown | |
| Novel_G001992 | uncharacterized protein | Unknown | |
| Novel_G003391 | uncharacterized protein | Unknown | |
| Novel_G004073 | uncharacterized protein | Unknown | |
| Novel_G004266 | uncharacterized protein | Unknown | |
| Novel_G001649 | uncharacterized protein | Unknown | |
| Novel_G000230 | uncharacterized protein | Unknown | |
| Novel_G002367 | uncharacterized protein | Unknown | |
| Novel_G003616 | uncharacterized protein | Unknown | |
| Novel_G003822 | uncharacterized protein | Unknown | |
| Novel_G003383 | uncharacterized protein | Unknown | |
| Novel_G002768 | uncharacterized protein | Unknown | |
| Novel_G004214 | uncharacterized protein | Unknown | |
| Novel_G000463 | uncharacterized protein | Unknown | |
| Novel_G001726 | uncharacterized protein | Unknown | |
| Novel_G003714 | uncharacterized protein | Unknown | |

^a^Gene ID is according to annotation of RefBeet reference genome (Dohm et al. 2014) that is available from <https://www.ncbi.nlm.nih.gov/genome/?term=Beta%20Vulgaris>. The ID started with “Novel” indicates it is a novel transcript identified in this study.
