# Supplementary figures and images for: Mechanism of Sugarbeet Seed Germination Enhanced by Hydrogen Peroxide
Source: Front Plant Sci. 2022 Apr 25;13:888519. doi: 10.3389/fpls.2022.888519 (PMC9082935; doi:10.3389/fpls.2022.888519)

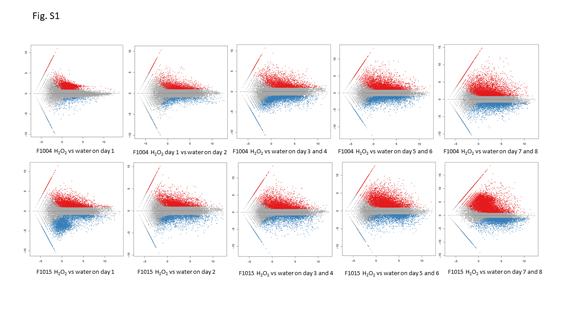

Supplement: Supplementary Figure 1 — Plots of transcriptome comparisons through Poisson Distribution method between the paired treatments of incubating seedballs of F1004 and F1015 in H2O2 solution and water. For each of plots, X-axis represents the value of log2 transformed mean expression level, Y-axis represents value of log2 transformed expression level fold change calculated from dividing level of H2O2 treatment with that in water control. Red dots represent up-regulated DEGs in H2O2 treatment, blue dots represent down-regulated ones, and gray points represent non-DEGs. [file Image_1.TIF]
